# Discovery of novel immune checkpoint regulators in a comprehensive library of human extracellular proteins

**DOI:** 10.1186/2051-1426-2-S3-P142

**Published:** 2014-11-06

**Authors:** Nathan A Sallee, Ernestine Lee, Andrew Rankin, Robert Halenbeck, Arthur Brace, Lewis T Williams, Brian Wong, W Michael Kavanaugh

**Affiliations:** 1 Five Prime Therapeutics, Inc., San Francisco, CA, USA

## 

Identification of novel targets in cancer immunotherapy is needed to address the significant number of patients that either do not respond to current therapies or encounter unacceptable toxicities. The discovery of such targets, including novel checkpoint regulators and the counter-receptors for previously "orphan" checkpoints, has been limited by a lack of a comprehensive collection of proteins suitable for functional screening and methods for assessing their function in high-throughput.

We have generated a comprehensive library of substantially all human extracellular proteins encompassing nearly every target for protein therapeutics. Our library contains more than 5700 proteins, including secreted protein ligands and the extracellular domains of membrane-bound receptors in soluble forms. In addition, we have developed in vitro and in vivo high-throughput protein screening platforms for discovering and validating new targets in a variety of disease areas. Our discovery platforms are uniquely suited for the discovery of novel targets in cancer immunotherapy. Based on domain structure, phylogeny and other factors, we identified a subset of 391 human proteins in our library that are enriched for possible cell surface-expressed immune regulatory proteins. These proteins were screened for the ability to modulate anti-CD3-stimulated human T cell proliferation. This screen identified a number of known checkpoint regulators, which validates the discovery platform and screening approach. In addition, we identified numerous novel co-inhibitory proteins - the majority of which have no published link to immune cell regulation. For a number of these proteins, we have demonstrated co-inhibitory activity in orthogonal assays, such as assays of cytokine production from purified T cell subpopulations and in mixed lymphocyte reaction assays. Further in vitro and in vivo characterization of these proteins is ongoing. These data demonstrate the power of our screening platform to discover potentially new therapeutic targets for cancer immunotherapy.

